# Lithium prescription patterns for bipolar disorder at a psychiatric hospital in KwaZulu-Natal

**DOI:** 10.4102/sajpsychiatry.v32i0.2576

**Published:** 2026-02-20

**Authors:** Ntibelleng N. Motebele, Vuyokazi Ntlantsana, Sibongile Mashaphu, Shamima Saloojee

**Affiliations:** 1 Department of Psychiatry, Faculty of Health Sciences, University of KwaZulu-Natal, Durban, South Africa

**Keywords:** lithium, bipolar disorder, South Africa, outpatient, mood stabiliser

## Abstract

**Background:**

Lithium is regarded as the gold standard in the treatment of bipolar disorder (BD) globally and is accordingly recommended as first-line treatment for BD in the South African public sector guidelines. However, there is a downward trend in the use of lithium internationally, with a paucity of research regarding lithium use in South Africa.

**Aim:**

This study aimed to investigate the prescription of lithium for outpatients with BD.

**Setting:**

Townhill Hospital, a tertiary pyschiatric hospital in Pietermaritzburg, KwaZulu- Natal.

**Methods:**

A retrospective review of the clinical records of outpatients aged 18 and above who were treated for BD was conducted from 01 August 2022 to 31 July 2024.

**Results:**

Of the 206 records that were reviewed, there were 137 (66.5%) females and 69 (33.5%) males with a median age of 37 years (interquartile range 30.0, 50.0). Only 13 (6.3%) of patients were taking lithium, all in combination with other medications. Most patients were prescribed oral antipsychotics (72.8%; *n* = 150) and anticonvulsant mood stabilisers (72.8%; *n* = 150), followed by antidepressants (55.8%; *n* = 115), alone or in combination with other psychotropics.

**Conclusion:**

The low prescription of lithium for BD in this study is concerning, despite the long-standing evidence for lithium’s efficacy and effectiveness in the literature. This highlights the need to remind clinicians about the South African BD guidelines.

**Contribution:**

The results of this study expand the existing literature on lithium prescribing patterns in BD internationally by contributing data on the prescription of lithium for BD in Africa.

## Introduction

Bipolar disorder (BD) is a common severe mental illness with an early age of onset that is characterised by recurrent mood episodes of varying duration and severity throughout the lifespan of the affected individual.^1,2^ Maintenance treatment is therefore essential in maintaining recovery and preventing future relapses.^3^ In a review of 15 randomised control trials, 61% of patients on placebo, and 39% on mood-stabilising drugs experienced a recurrence of symptoms within 1.9 years.^4^ Relapses and recurrences in BD may be result from non-adherence to medication, ineffective treatment, inadequate treatment, illness progression,^2^ cultural beliefs, stigma and access to care particularly in low- and middle-income countries (LMICs).^5^

There is good evidence that illness recurrence and relapse are associated with social impairment, occupational impairment, cognitive impairment and even suicide resulting in a high burden of disability for affected individuals and their families.^6,7^ Several medications including lithium, anticonvulsant mood stabilisers and antipsychotics, alone or in combination are effective in preventing recurrent episodes of the disorder.^2,5,8^ Generally, medications that have been effective during episodes of acute mania or bipolar depression should be continued in the maintenance phase.^7^ Medication adjustment during the acute phase is generally indicated, followed by maintenance treatment, this being essential for sustaining recovery and preventing future relapses.^3^

In the public sector of South Africa (SA), the National Department of Health essential medicine list (NDOH EML) selectors, mindful of the effectiveness and affordability of lithium, recommend lithium as the first-line treatment in the acute and maintenance phases of BD.^9^ This recommendation is based on an extensive body of evidence gathered over seven decades since lithium was first reported to be an effective medication for BD.^10^ Mood-stabilising anticonvulsant medications are second-line treatments, with antipsychotics recommended as third- and fourth-line agents for patients who do not respond to lithium, experience side effects, or are of childbearing age.^9^ Apart from lithium’s antimanic properties, and efficacy in preventing future episodes of BD I and BD II,^1,11^ lithium has also been shown to reduce suicide in vulnerable patients.^12^ Furthermore, there is also evidence that lithium has neuroprotective effects.^13^ Lithium is thus considered the gold standard in the treatment of BD.^14,15,16^

Despite the proven effectiveness of lithium, research found a lithium paradox,^15^ this being that, although there is a solid evidence base for the utility of lithium in BD there is a decline in its prescriptions internationally.^17^ Previous studies show that lithium prescription rates range from 3.3% to 84%,^18^ with regional and even intra-country variation.^19^ In a review of 55 studies over 56 years, lithium prescription frequencies were recorded for countries across North America, Europe and Asia, but not Africa.^18^ Data from Africa was also not included in a recent Global Bipolar Cohort collaborative network study on pharmacotherapy in BD.^20^

This study was motivated by the limited information regarding the selection of medicines for BD in the public sector generally,^21^ and lithium specifically.

The aim of this study, therefore, was to investigate lithium prescription patterns in the pharmacological treatment of patients with bipolar disorder at a specialised psychiatric hospital. The objectives of the study were (1) to determine the proportions of patients who were prescribed lithium alone, and in combination with other psychotropics in the maintenance phase of bipolar disorder, (2) to determine the prevalence of lithium monotherapy vs. combination therapy in patients with bipolar disorder, and (3) to determine if there was an association between any sociodemographic or clinical factors and the prescription of lithium.

## Research methods and design

### Study design and setting

This study was a descriptive, observational retrospective chart review. It was conducted at Townhill Hospital (THH), a specialised psychiatric hospital in KwaZulu-Natal (KZN). Townhill Hospital is a 282 bedded specialised psychiatric hospital that serves the uMgungundlovu District. Patients are referred from primary health clinics, stand-alone community psychiatric clinics, district hospitals, regional hospitals, private medical practitioners and psychiatrists in the uMgungundlovu district of KZN province. The follow-up outpatient clinic provides a tertiary-level referral service for patients who require more specialised care. The clinic does not accept bookings for acutely ill patients needing psychiatric care and treatment.

### Study population and sampling strategy

The study population consisted of consecutive adult men and women over the age of 18 years who were treated for a BD in the outpatient clinic at THH. The diagnosis of BD was extracted from the clinical records of patients attending the clinic, and all diagnoses were made by psychiatric registrars in accordance with Diagnostic and Statistical Manual of Mental Disorders, Fifth Edition (DSM 5) criteria.^22^ Registrars were supervised by full-time specialist psychiatrists in the clinic.

The clinical records of patients who attended the clinic were reviewed over a 2-year period from 01 August 2022 to 01 August 2024. No new referrals were included in the sample; the sample consisted of maintenance treatment for follow-up patients only. The records of patients who attended the outpatient clinic on more than one occasion were only reviewed on the first attendance. Incomplete records of patients treated for BD were excluded.

### Measures

Data were extracted using a questionnaire specifically designed for the study. The information collected included sociodemographic, clinical and medication-related details. Valproate, lamotrigine and carbamazepine were categorised as anticonvulsant mood stabilisers. Second-generation antipsychotics were olanzapine, quetiapine, risperidone, clozapine and aripiprazole, while antidepressants included fluoxetine, citalopram, amitriptyline, bupropion and venlafaxine. The data were captured onto an electronic REDCap database,^23^ which is a secure, password-protected and web-based research database offered by the University of KwaZulu-Natal (UKZN).

### Statistical methods

Statistical analyses were conducted using Stata v18. For descriptive analyses, frequencies, mean, median and standard deviation were used to describe continuous data. Frequencies and percentages were used to describe categorical data. Pearson’s correlation coefficient was used to test for association between sociodemographic, clinical factors and the prescription on individual classes of medication.

### Ethical considerations

Ethical approval to conduct the study was obtained from the Biomedical Research Ethics Committee (reference number BREC/00007320/2024) of the University of KwaZulu-Natal. Permission to access clinical records was obtained from the KZN Department of Health and THH ethics committee.

## Results

The clinical records of 215 patients who were treated in the follow-up clinic at the study hospital in the maintenance phase of BD over the study period were examined; two records were excluded because of missing information. A further seven patients who had relapsed and were thus classified as being in the acute phase of illness were excluded.

Of the 206 patients who were included in the analysis, 137 (66.5%) were females and 69 (33.5%) were males, with a median age of 37 years (interquartile range [IQR] 30.0, 50.0). Most of the participants were single (61.7%), of white ethnicity (47.8%), unemployed (77.7%), and were referred from primary or secondary level care in the province (52.9%). More patients were diagnosed with bipolar II disorder (56.3%) than bipolar I disorder (43.7%); the median age of onset was 24 years (IQR 19.0, 33.0) with prominent psychiatric (69.9%) and medical comorbidity (41.7%) (Table 1).

**TABLE 1:** Sociodemographic and clinical characteristics (*N* = 206).

Variable	*n*	%	Median	IQR
**Sex**				
Female	137	66.5	-	-
Male	69	33.5	-	-
Age	-	-	37.0	30.0, 50.0
**Marital status**				
Single	127	61.7	-	-
Married	46	22.3	-	-
Other	33	16.0	-	-
**Ethnicity**				
Black people	55	27.1	-	-
Indian people	31	15.3	-	-
White people	97	47.8	-	-
Mixed race people	20	9.9	-	-
**Level of education**				
Nil	1	0.5	-	-
Remedial school	3	1.5	-	-
Mainstream	130	63.7	-	-
Tertiary	70	34.3	-	-
**Employment status**				
Unemployed	160	77.7	-	-
Employed	46	22.3	-	-
**Disability grant**				
No	149	72.3	-	-
Yes	57	27.7	-	-
**Subtype of bipolar disorder**				
Type I	90	43.7	-	-
Type II	116	56.3	-	-
**Source of referral**				
Public	109	52.9	-	-
Private	72	35.0	-	-
Self	7	3.4	-	-
Other	18	8.7	-	-
Age at onset	-	-	24.0	19.0, 33.0
Any comorbid psychiatric illness	144	69.9	-	-
Any comorbid medical illness	86	41.7	-	-
Lifetime tobacco use	109	52.9	-	-
Lifetime alcohol use	111	54.1	-	-
Lifetime cannabis	71	34.5	-	-

IQR, interquartile range.

Table 2 shows that a range of medication was prescribed for patients in the maintenance phases of both manic and depressive episodes. Only 6.3% (*n* = 13) patients were taking lithium, with 72.8 % (*n* = 150) on anticonvulsant mood stabilisers, 8.3% (*n* = 17) on first-generation antipsychotics, 71.8% (*n* = 148) on second-generation antipsychotics and 55.8% (*n* = 115) on antidepressants alone or in combination with other psychotropics. Polypharmacy was the rule, with only 19.9% of the participants taking one drug, 42.7% (*n* = 88) taking two drugs and 37.4% (*n* = 77) taking three or more drugs.

**TABLE 2:** Utilisation of drugs by episode.

Drugs	All		Depressed		Manic		*P*-value	Test
	*n*	%	*n*	%	*n*	%		
*N*	206	-	133	64.6	73	35.4	-	-
**Lithium**	-	-	-	-	-	-	0.015	Pearson’s Chi-squared
No	193	93.7	127	95.5	66	90.4	-	-
Yes	13	6.3	6	4.5	7	9.6	-	-
**Anticonvulsant MS**	-	-	-	-	-	-	0.091	Pearson’s Chi-squared
No	56	27.2	31	23.3	25	34.2	-	-
Yes	150	72.8	102	76.7	48	65.8	-	-
**FGA**	-	-	-	-	-	-	0.062	Fisher’s exact
No	202	98.1	131	98.5	71	97.3	-	-
Yes	4	1.9	2	1.5	2	2.7	-	-
**SGA**	-	-	-	-	-	-	0.072	Pearson’s Chi-squared
No	58	28.2	43	32.3	15	20.5	-	-
Yes	148	71.8	90	67.7	58	79.5	-	-
**LAI Antipsychotic**	-	-	-	-	-	-	0.002	Fisher’s exact
No	193	93.7	130	97.7	63	86.3	-	-
Yes	13	6.3	3	2.3	10	13.7	-	-
**Antidepressants**	-	-	-	-	-	-	< 0.001	Pearson’s Chi-squared
No	91	44.2	38	28.6	53	72.6	-	-
Yes	115	55.8	95	71.4	20	27.4	-	-
**Benzodiazepines**	-	-	-	-	-	-	0.015	Fisher’s exact
No	185	89.8	116	87.2	69	94.5	-	-
Yes	21	10.2	17	12.8	4	5.5	-	-
**Number of drugs**	-	-	-	-	-	-	0.011	Pearson’s Chi-squared
Monotherapy	41	19.9	19	14.3	22	30.1	-	-
2 drugs	88	42.7	57	42.9	31	42.5	-	-
3 or more drugs	77	37.4	57	42.9	20	27.4	-	-

Anticonvulsant MS, Anticonvulsant mood stabilisers; FGA, First generation antipsychotic; SGA, Second generation antipsychotic; LAI, Long-acting injectable.

Lithium prescription patterns (Table 3) show that, of the 13 patients who were prescribed lithium, not a single patient was prescribed lithium alone, with 61.5% taking lithium combined with one other medication and 38.5% taking lithium combined with two or more medications. The most frequently prescribed medication in combination with lithium was antipsychotic medication (30.7%), followed by anticonvulsant mood stabilisers (15.4%) and antidepressants (15.4%). In addition, six patients (46.1%) were commenced on lithium prior to being referred to TH, while lithium was initiated in seven patients (53.9%) at THH. Dosages ranged between 400 and 1200 mg/day for all patients, with seven (53.9%) on a single daily dose regimen and the remaining six (46.1%) on a twice daily dose.

**TABLE 3:** Lithium prescription patterns.

Lithium combined with	*n*	%
Anticonvulsant mood stabilisers (MS)	2	15.4
Antipsychotics	4	30.7
Antidepressants	2	15.4
Anticonvulsant MS and antipsychotics	3	23.1
Anticonvulsants MS and antidepressants	1	7.7
Anticonvulsant MS and antipsychotics and antidepressants	1	7.7

The only significant association between lithium prescription and sociodemographic and clinical variables was comorbid psychiatric illness (*p* = 0.05) (Table 4).

**TABLE 4:** Predictors of lithium utilisation by sociodemographic and clinical variables.

Characteristic	Lithium								*P*-value	Test
	No				Yes					
	*n*	%	Median	IQR	*n*	%	Median	IQR
*N*	193	93.6	-	-	13	6.4	-	-	-	-
**Sex**	-	-	-	-	-	-	-	-	1.00	Fisher’s exact
Female	128	66.3	-	-	9	69.2	-	-	-	-
Male	65	33.7	-	-	4	30.8	-	-	-	-
Age	-	-	37.0	30.0, 50.0	-	-	42.0	31.0, 45.0	0.70	Wilcoxon rank-sum
**Marital status**	-	-	-	-	-	-	-	-	0.66	Fisher’s exact
Single	120	62.2	-	-	7	53.8	-	-	-	-
Married	43	22.3	-	-	3	23.1	-	-	-	-
Other	30	15.5	-	-	3	23.1	-	-	-	-
**Ethnicity**	-	-	-	-	-	-	-	-	0.46	Fisher’s exact
Black people	50	26.3	-	-	5	38.5	-	-	-	-
Indian people	28	14.7	-	-	3	23.1	-	-	-	-
White people	92	48.4	-	-	5	38.5	-	-	-	-
Mixed race people	20	10.5	-	-	0	0.0	-	-	-	-
**Level of education**	-	-	-	-	-	-	-	-	0.10	Fisher’s exact
Nil	1	0.5	-	-	0	0.0	-	-	-	-
Remedial school	126	65.6	-	-	4	33.3	-	-	-	-
Mainstream	3	1.6	-	-	0	0.0	-	-	-	-
Tertiary	62	32.3	-	-	8	66.7	-	-	-	-
**Employment status**	-	-	-	-	-	-	-	-	0.17	Fisher’s exact
Unemployed	152	78.8	-	-	8	61.5	-	-	-	-
Employed	41	21.2	-	-	5	38.5	-	-	-	-
**Disability grant**	-	-	-	-	-	-	-	-	0.76	Fisher’s exact
No	140	72.5	-	-	9	69.2	-	-	-	-
Yes	53	27.5	-	-	4	30.8	-	-	-	-
**Subtype of bipolar disorder**	-	-	-	-	-	-	-	-	0.45	Pearson’s Chi-squared
Type I	83	43.0	-	-	7	53.8	-	-	-	-
Type II	110	57.0	-	-	6	46.2	-	-	-	-
**Source of referral**	-	-	-	-	-	-	-	-	0.58	Fisher’s exact
Public	101	52.3	-	-	8	61.5	-	-	-	-
Private	69	35.8	-	-	3	23.1	-	-	-	-
Self	7	3.6	-	-	0	0.0	-	-	-	-
Other	16	8.3	-	-	2	15.4	-	-	-	-
Age at onset	-	-	24.0	19.0, 34.0	-	-	23.0	19.0, 28.0	0.40	Wilcoxon rank-sum
**Any comorbid psych illness**	-	-	-	-	-	-	-	-	0.054	Pearson’s Chi-squared
No psychiatric comorbidities	55	28.5	-	-	7	53.8	-	-	-	-
Has psychiatric comorbidities	138	71.5	-	-	6	46.2	-	-	-	-
**Any comorbid medical illness**	-	-	-	-	-	-	-	-	1.00	Fisher’s exact
No medical comorbidities	112	58.0	-	-	8	61.5	-	-	-	-
Has medical comorbidities	81	42.0	-	-	5	38.5	-	-	-	-
**Lifetime tobacco**	-	-	-	-	-	-	-	-	0.94	Pearson’s Chi-squared
No	91	47.2	-	-	6	46.2	-	-	-	-
Yes	102	52.8	-	-	7	53.8	-	-	-	-
**Lifetime alcohol**	-	-	-	-	-	-	-	-	0.39	Fisher’s exact
No	90	46.9	-	-	4	30.8	-	-	-	-
Yes	102	53.1	-	-	9	69.2	-	-	-	-
**Lifetime cannabis**	-	-	-	-	-	-	-	-	1.00	Fisher’s exact
No	126	65.3	-	-	9	69.2	-	-	-	-
Yes	67	34.7	-	-	4	30.8	-	-	-	-

IQR, interquartile range.

## Discussion

The main finding of this study is that of the 206 outpatients being treated for BD, with a median age of 37 years, only 13 patients (6.3%) were taking lithium, with not a single patient being prescribed lithium alone. The study also confirmed a preference for the prescription of oral antipsychotics and mood stabilisers, with almost three-quarters of the patients taking them alone or in combination with other medications for the treatment of BD at the study site. Antidepressants were prescribed for over half of the patients.

The proportion of patients on lithium in this study (6.3%) is compared to rates of 43.6% from North America, 36.4% from Europe, and 20.0% from Asia published in a recent scoping review of international trends of lithium use in bipolar disorder.^18^ The proportion of patients on lithium in this study is also significantly lower than the proportion of 29% reported in a global cohort of patients with BD.^20^ Evidence from Africa and SA is limited. The prescription of lithium in this study is lower than that reported in a study conducted in Gauteng, where lithium was prescribed in 34.3% of patients with BD.^21^ However, our result is supported by a recent study at another psychiatric hospital in KZN that found that valproate was combined with lithium in only 1.3% of patients.^24^ A study from Nigeria also found that only 10.89% of psychiatrists, registrars, and medical officers working in psychiatric facilities reported that they prescribed lithium for BD.^25^

Reasons for selecting lithium from the list of medications licensed for the treatment of BD are multifactorial, and include the presence of good predictors of clinical response,^26^ comorbid medical and psychiatric illnesses,^27^ side effect concerns including the need for laboratory monitoring,^28^ SA public sector cost,^21^ clinicians’ training,^29^ professional judgement or preferences,^2^ and possible risk of suicide.^30^

Mindful of these factors, possible explanations for the low utilisation of lithium in this study compared to the other medication classes include the presence of comorbid illnesses such as HIV, because KZN had the highest number of people living with HIV in SA in 2022.^31^ It is possible that doctors avoided prescribing lithium given that individually the renal adverse effects of lithium,^28^ HIV itself, and antiretroviral medication^32^ are well known, and that these drug interactions cumulatively could increase the potential for nephrotoxicity.

The narrow therapeutic index and teratogenic adverse events of lithium may have also contributed to the low prescription rate.^28^ Stable patients with BD are routinely down referred from the psychiatric hospital where this study was conducted to community mental health clinics. The need for repeated serum lithium levels at outpatient, primary and district-level mental healthcare services is problematic, because these levels of care are poorly resourced and understaffed, with a large treatment gap for mental illnesses at these levels in SA.^33^ It is unlikely, however, that lithium was avoided in women of childbearing age because there was no significant difference in the proportion of women taking lithium compared to those who are not on lithium.

A study from Italy found that only half of early-career psychiatrists reported that their knowledge regarding lithium prescriptions was adequate. The authors recommended that the training programme for psychiatrists should include education on lithium specifically.^29^ The clinic in this study is run by registrars in psychiatry, and it is possible that the amount of time dedicated to lithium for the management of BD in their postgraduate training is insufficient.

Lithium was only prescribed in combination with other medications in this study. This is a common practice to reduce relapse risk.^34^ However, evidence for the superiority of lithium combination therapy over monotherapy is equivocal. In a secondary analysis of 370 outpatients with BD, lithium combined with an antipsychotic or an anticonvulsant did not show a superior clinical response to lithium alone.^35^ The BALANCE trial, a large randomised open-label trial found that lithium combined with valproate was no better or worse than lithium alone.^36^ However, a population-based cohort study from Sweden reported that lithium combination therapy (with quetiapine and valproate) was associated with a lower risk of treatment failure compared to lithium monotherapy.^34^ Overall, therefore it is possible that lithium was combined with other mood-stabilising medications to improve response rates to lithium.

Of concern is the lack of concordance (less than 10%) in this study with the South African public sector NDOH EML guidelines.^9^ Previous research shows that international guideline concordance rates for BD vary widely ranging from 16% to 80%, with most studies in the 50% – 80% range.^37^ It can be argued that the true concordance in this study is higher because the NDOH EML guidelines follow a hierarchical structure, and it is possible that many patients in this study currently on antipsychotics or anticonvulsants did not respond to lithium. However, the median age of the patients on lithium (42 years) was higher than those not on lithium (37 years), and lithium was combined with two or three other agents in most patients, making it more likely that the patients on lithium were difficult to treat, or that lithium was used as a last resort rather than a first-line agent as recommended by the SA guidelines. Furthermore, even taking into account that patients not on lithium currently may have been poor responders to lithium previously will not explain the low utilisation in this study, because studies have shown that between 40% and 50% of patients do not respond sufficiently to lithium.^38^

Other barriers to the appropriate pharmacological management of BD in SA and other LMIC include missed BD diagnoses, mental health services that are inadequate for the needs of the population, the limited number of medications available for bipolar disorder on the essential medicines list of SA (EML),^5^ and frequent problems with the supply and procurement of essential medicines (medicine stockouts) in SA.^31^

### Limitations

Firstly, the limitations of this study include a lack of information regarding previous prescriptions for BD (at the referral sites), so we could not identify changes in treatment. Secondly, the findings of the study cannot be generalised to other hospitals in KZN or other provinces in SA. Thirdly, the retrospective study design limits the accuracy and completeness of the findings because of the reliance on information captured by many different clinicians in the clinical records over time. Lastly, the small number of patients taking lithium was not adequate to identify predictors of lithium response, and we did not collect information regarding adverse events or the monitoring of serum lithium levels in the patients who were taking lithium.

## Conclusion

The low prescription of lithium in this study confirms that lithium is a ‘forgotten drug’ at THH.^39^ The low utilisation of lithium is highlighted against a background of ample evidence in the literature for the efficacy and effectiveness of lithium in the acute and maintenance phases of BD for more than 50 years.^9,14,40^

### Recommendations

Initiatives to improve lithium prescription should include educational programmes to dispel the myths associated with lithium prescriptions,^41^ because there is a suggestion that the adverse event profile of lithium is exaggerated.^42^ Undertaking studies to investigate the prevalence of lithium side effects in patients living with HIV in KZN, conducting surveys among early and late-career psychiatrists in SA to determine reasons for their reluctance to prescribe lithium and establishing specialised BD clinics^17^ are further recommendations.
